# Meta‐Analysis of Thermal Versus Pulse Field Ablation for Pulmonary Vein Isolation Durability in Atrial Fibrillation: Insights From Repeat Ablation

**DOI:** 10.1002/clc.70151

**Published:** 2025-05-28

**Authors:** Jin‐Cheng Chen, Yin‐Jun Mao, Qun‐Ying Huang, Zhen‐Dong Cheng, Shao‐Bin He, Qiu‐Xia Xu, Yin Zhang

**Affiliations:** ^1^ Laboratory of Clinical Pharmacy, Department of Pharmacy The Second Affiliated Hospital of Fujian Medical University Quanzhou China; ^2^ Department of Pharmacy First Affiliated Hospital of Fujian Medical University Fuzhou China; ^3^ Department of Pharmacy, National Regional Medical Center, Binhai Campus of the First Affiliated Hospital Fujian Medical University Fuzhou China; ^4^ Department of Cardiology First Affiliated Hospital of Fujian Medical University Fuzhou China; ^5^ Department of Cardiology The Second Affiliated Hospital of Fujian Medical University Quanzhou China

**Keywords:** atrial fibrillation, pulsed field ablation, PVI durability, thermal ablation

## Abstract

**Purpose:**

Pulsed field ablation (PFA) represents an innovative technique for pulmonary vein isolation (PVI), exhibiting promising efficacy and safety in initial clinical studies. However, the long‐term durability of PVI and reconnection patterns following PFA are not as well‐characterized compared to those associated with thermal energy sources such as cryoballoon ablation (CBA) or radiofrequency ablation (RFA). The objective of this study is to compare the durability of lesions resulting from repeat ablation after index PVI using PFA versus thermal ablation (TA).

**Methods:**

We conducted a comprehensive search of multiple databases to identify relevant studies published before January 2025. PVI durability was assessed on a per patient and per vein level.

**Results:**

This study included 14 trials involving a total of 7,367 patients. PFA demonstrated a trend toward reduced rates of repeat ablation in comparison to TA (OR 0.77; 95% CI, 0.59–1.00). Durability of PVI per patient (OR 1.42; 95% CI, 0.92–2.19) or per vein (OR 1.42; 95% CI, 0.92–2.19) were similar after PFA and TA. The most common site of reconnection was the right inferior PV for both PFA and TA (39.7% and 38.1%, respectively). Subgroup analyses demonstrated that PVI durability per vein was significantly higher with PFA compared to RFA (OR 1.32; 95% CI, 1.03–1.70).

**Conclusion:**

At the time of repeat ablation, PFA exhibited a trend toward a reduced incidence of repeat procedures compared to CBA or RFA. PFA is comparable to CBA in achieving durable isolation of all veins but demonstrates superiority over RFA.

**Abbreviations**
AF: atrial fibrillation
ATa: atrial tachyarrhythmia
CBA: cryoballoon ablation
LCPV: left common PV
LIPV: left inferior PV
LSPV: left superior PV
PAF: paroxysmal AF
PFA: pulsed field ablation
PVI: pulmonary vein isolation
RCT: randomized controlled trial
RFA: radiofrequency ablation
RIPV: right inferior PV
RSPV: right superior PV
TA: thermal ablation

## 1 Introduction

Pulmonary vein isolation (PVI) remains the primary objective in atrial fibrillation (AF) ablation [1]. Emerging evidence supporting the efficacy of rhythm control strategies in improving outcomes for AF patients [2], along with recent guideline updates, has resulted in a growing number of patients being referred for this procedure [3]. In recent years, technological advancements have concurrently enabled a comprehensive comparison between established techniques, such as cryoballoon ablation (CBA) [4] single‐shot devices and contact force‐sensing irrigated radiofrequency ablation (RFA) [5] catheters, and novel technologies that are highly effective and well‐tolerated, as recently highlighted in the literature. Pulsed field ablation (PFA) represents an innovative energy modality that induces electroporation with minimal thermal alterations in tissue compared to CBA or RFA, thereby positioning it as a highly promising technique for PVI [6]. Initial safety studies suggest a reduced complication rate associated with PV stenosis, atrioesophageal fistula, and phrenic nerve palsy compared to RFA [7]. The efficacy of this approach is comparable to thermal ablation (TA) strategies for PVI, as demonstrated in the first randomized controlled trial (RCT) examining this issue using time to first recurrence of AF as the primary endpoint [8]. Consistent findings were reported in a recent meta‐analysis encompassing 1880 patients [9].

Electrical reconnection of previously isolated PVs remains the most frequently observed phenomenon following PVI procedures, whether conducted using TA or PFA [10, 11]. During repeat procedures, the durability of PVI exhibits considerable variability, with reported estimates ranging from 3% to 53% following TA (Table S1). Incomplete lesion formation during TA is often linked to several factors, including suboptimal low nadir and mean temperatures [12], mismatches between balloon size and patient anatomy [13], insufficient contact force [14], and changes in tissue impedance [15]. For lesion formation using PFA, tissue contact remains crucial, and lesion size is influenced by contact force [16]. However, these patient‐ and application‐specific factors may not be as pertinent to PFA, given that the technology's selective targeting of cardiomyocytes suggests a broader therapeutic window for energy delivery. Therefore, PVI durability may be significantly enhanced during the repeat procedures after PFA compared with TA. However, the available data concerning the durability of PVI are limited, especially when compared to TA. Thus, this study aims to compare the lesion durability on a per vein and per patient level during the repeat procedure after index PFA and TA.

## 2 Methods

### 2.1 Database Search and Selection Criteria

A systematic search was conducted across the EMBASE, PubMed, Cochrane Library, and ClinicalTrials.gov databases to identify relevant trials comparing PFA and TA for repeat procedures following an initial ablation for AF, from the inception of these databases until January 2025. The following keywords were utilized: “pulsed field ablation,” “PVI durability,” “cryoballoon ablation,” “radiofrequency ablation,” “thermal ablation,” and “atrial fibrillation.” Furthermore, we conducted a manual review of the references cited in the retrieved articles and examined proceedings from recent major cardiovascular conferences to ensure the inclusion of any relevant studies that might have been overlooked during the initial literature search.

The inclusion criteria for eligible studies were as follows: (1) Studies comparing outcomes between patients who underwent a subsequent repeat procedure for recurrent atrial tachyarrhythmia (ATa) after their first PFA and TA (CBA or RFA); (2) Studies that reported relevant results; and (3) Research published in full in the English language. Case reports, case series, nonhuman studies, reviews, and editorials were excluded from this analysis.

### 2.2 Data Extraction and Quality Assessment

The authors independently extracted pertinent data from the eligible studies and achieved consensus on all aspects. Any discrepancies were addressed through discussion and resolved amicably. Extract the following information from each trial: participant characteristics (mean age, proportion of males, coronary artery disease, body mass index, diabetes mellitus, hypertension, dyslipidemia, transient ischemic attack/stroke, and left ventricular ejection fraction and left atrium diameter) as well as study characteristics (study author, publication year, study population, sample size, intervention strategy, assessment methods, and follow‐up period).

The Cochrane Risk of Bias Tool was applied for RCTs, whereas the Newcastle‐Ottawa Scale was used to evaluate the risk of bias in observational studies.

### 2.3 Study Endpoints

The primary endpoints of this study included the evaluation of PVI durability and the rate of repeat procedures. PVI durability was assessed at both the per patient level, by determining the proportion of patients achieving complete PVI, and at the per vein level, by quantifying the number of successfully isolated veins. The repeat procedure rate was calculated as the ratio of patients who underwent repeat ablation to the total number of patients who initially received ablation. Secondary endpoints encompassed the incidence rates of individual PV reconnection (right superior PV [RSPV], right inferior PV [RIPV], left superior PV [LSPV], and left inferior PV [LIPV]).

### 2.4 Statistical Analysis

The odds ratio (OR) with 95% confidence intervals (CI) was computed for dichotomous variables. Heterogeneity was evaluated using the Cochrane I^2^ statistic and categorized as follows: low (I^2^ ≤ 25%), moderate (I^2^ > 25% and < 75%), or high (I^2^ ≥ 75%) [17]. In instances of low heterogeneity, a fixed‐effect model was applied; otherwise, a random‐effects model was utilized. We performed a sensitivity analysis to explore the sources of heterogeneity by sequentially excluding one study at a time. Additionally, we had pre‐planned subgroup analyses based on the type of energy source (CBA or RFA). However, due to the inclusion of only one RCT, conducting subgroup analysis based on study design was not feasible. Visualization results were evaluated through funnel plot symmetry to assess potential publication bias. Statistical significance was established at a two‐tailed P value less than 0.05. All analyses were conducted using RevMan software version 5.3.5.

## 3 Results

### 3.1 Search Results and Study Characteristics

**FIGURE 1:**
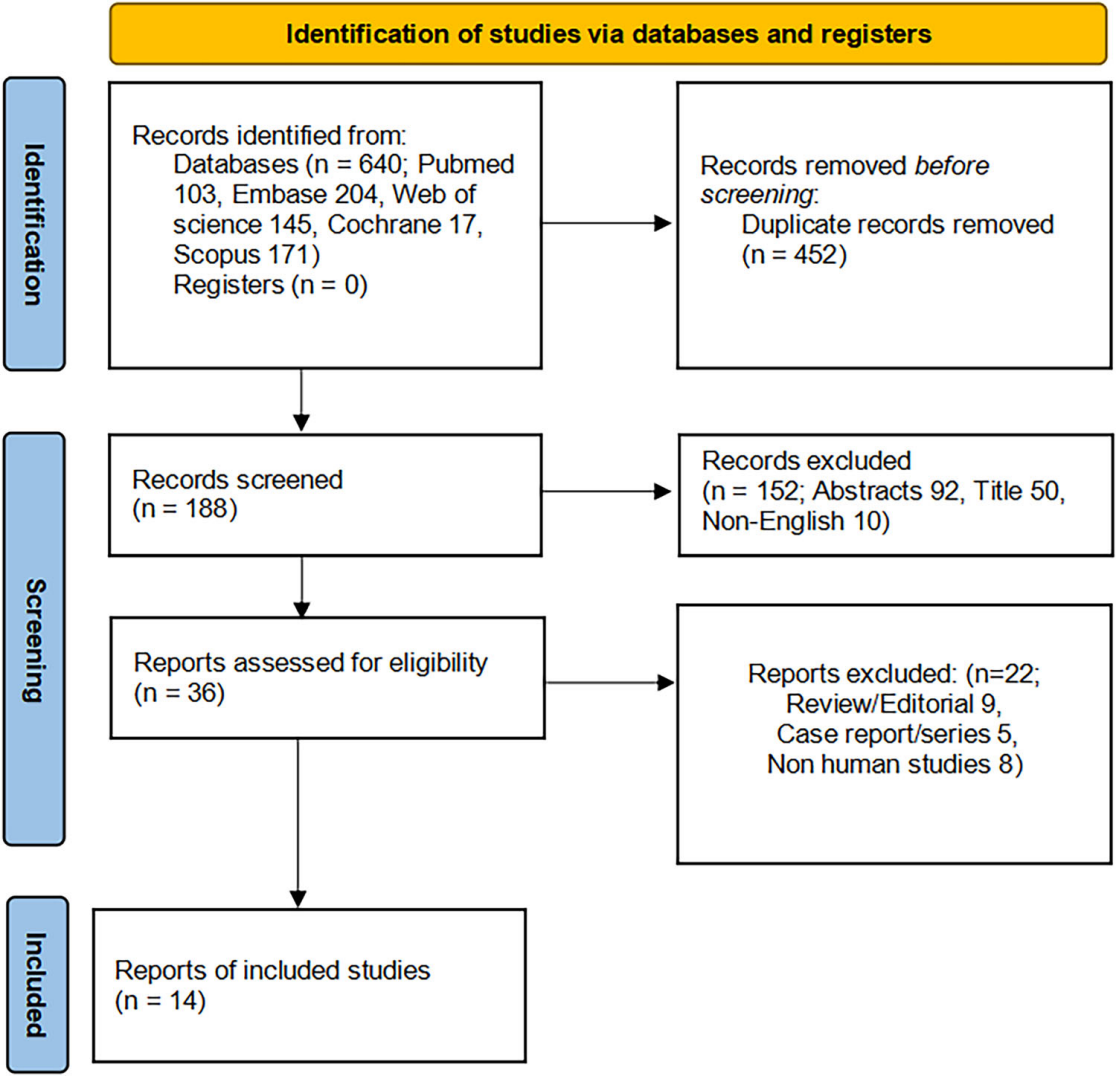
Flowchart illustrating the study selection process according to the PRISMA guidelines.

The PRISMA flow diagram offers a systematic overview of the literature selection process (Figure 1). This study ultimately encompassed 14 trials involving a total of 7367 participants (2597 in the PFA group and 4770 in the TA group, which included 65% CBA and 35% RFA) published from 2023 to 2024 [8, 18, 19, 20, 21, 22, 23, 24, 25, 26, 27, 28, 29, 30]. The trials comprised 13 observational studies, including five prospective cohorts [18, 23, 24, 25, 26] and eight retrospective cohorts [19, 20, 21, 22, 27, 28, 29, 30], as well as one RCT [8]. The index procedure baseline characteristics of the included studies are presented in Tables S2 and S3. Among the included studies, four did not provide per patient PVI durability data [18, 22, 24, 30], three failed to report per vein PVI durability data [21, 22, 30], and two only reported redo procedure rates without PVI durability data for either per patient or per vein [22, 30]. Only one study reported the baseline characteristics of patients undergoing repeated ablation procedures in two groups [25].

Regarding the RCT conducted by Reddy et al., [8] it exhibited a low risk of bias in selection (random sequence generation and allocation concealment), performance (blinding of participants and personnel), detection (blinding of outcome assessment), attrition (comparatively complete outcome data), and reporting bias (all prespecified outcomes were reported in accordance with the study protocol) (Table S4). Of the 13 cohort studies, two were rated as moderate quality due to inadequate adjustment for confounding factors [25, 30], while the remaining studies were rated as high quality (Table S5).

### 3.2 The Primary Endpoints

**FIGURE 2:**
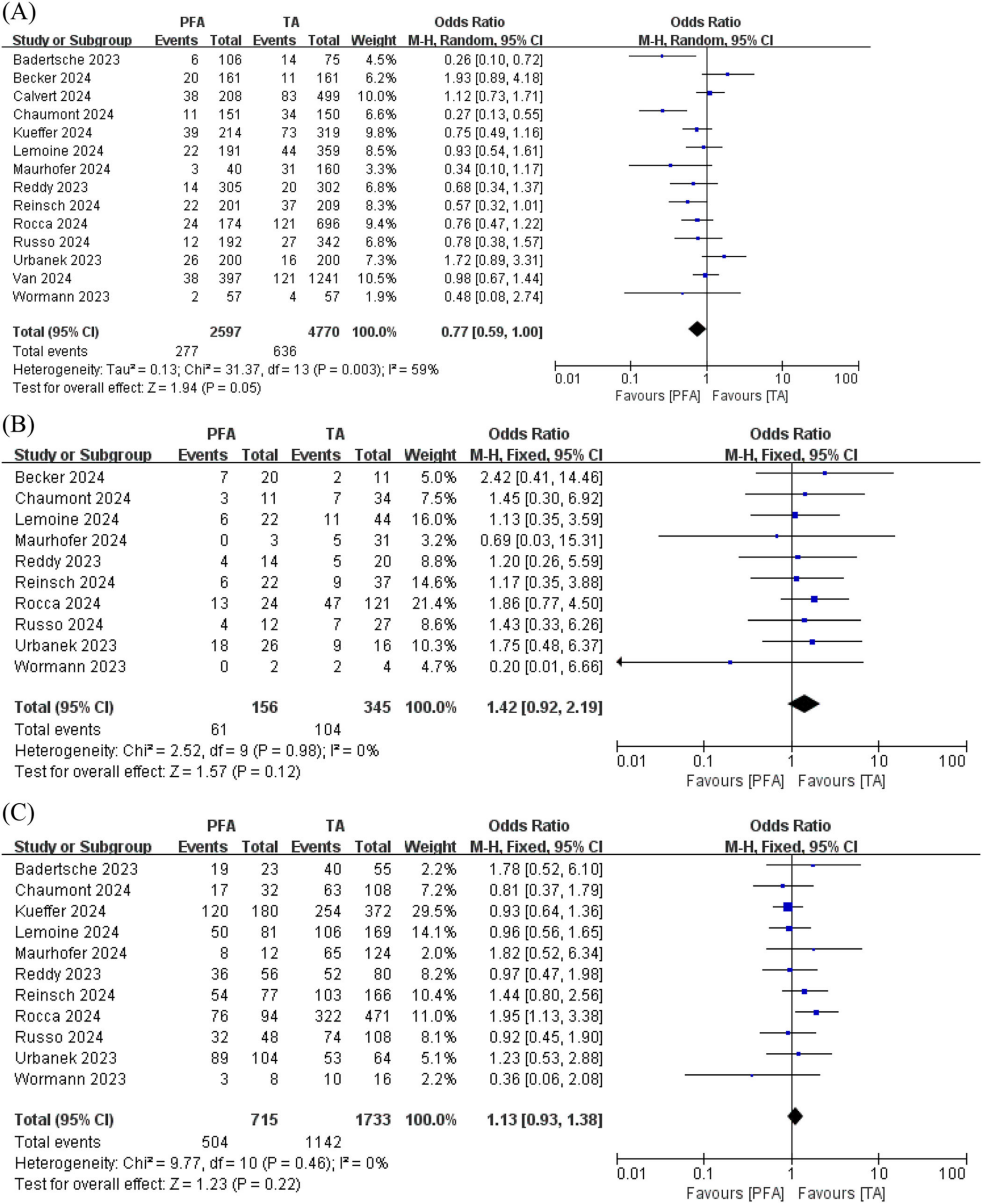
The forest plot displays the primary endpoints when comparing PFA and TA for atrial fibrillation. (A) the rate of repeat procedures, (B) PVI durability per patient, (C) PVI durability per vein. CI, confidence interval; PFA, pulsed field ablation; PVI, pulmonary vein isolation; TA, thermal ablation.

Repeat ablation procedures are seldom necessary after index PFA (277/2597; 10.7%) or TA (636/4770; 13.3%). Moreover, PFA demonstrated a trend toward reduced rates of repeat ablation procedures in comparison to TA (OR 0.77; 95% CI, 0.59–1.00; *p* = 0.05; I^2^ = 59%; Figure 2A).

The durability of per patient PVI was comparable between PFA (61/156; 39.1%) and TA (104/345, 30.1%) (OR 1.42; 95% CI, 0.92–2.19; *p* = 0.12; I^2^ = 0%; Figure 2B). Similarly, in the per vein analysis, the durability was equivalent for PFA (504/715, 70.5%) and TA (63/169, 65.9%) (OR 1.13; 95% CI, 0.93–1.38; *p* = 0.22; I^2^ = 0%;; Figure 2C).

### 3.3 The Secondary Endpoints

**FIGURE 3:**
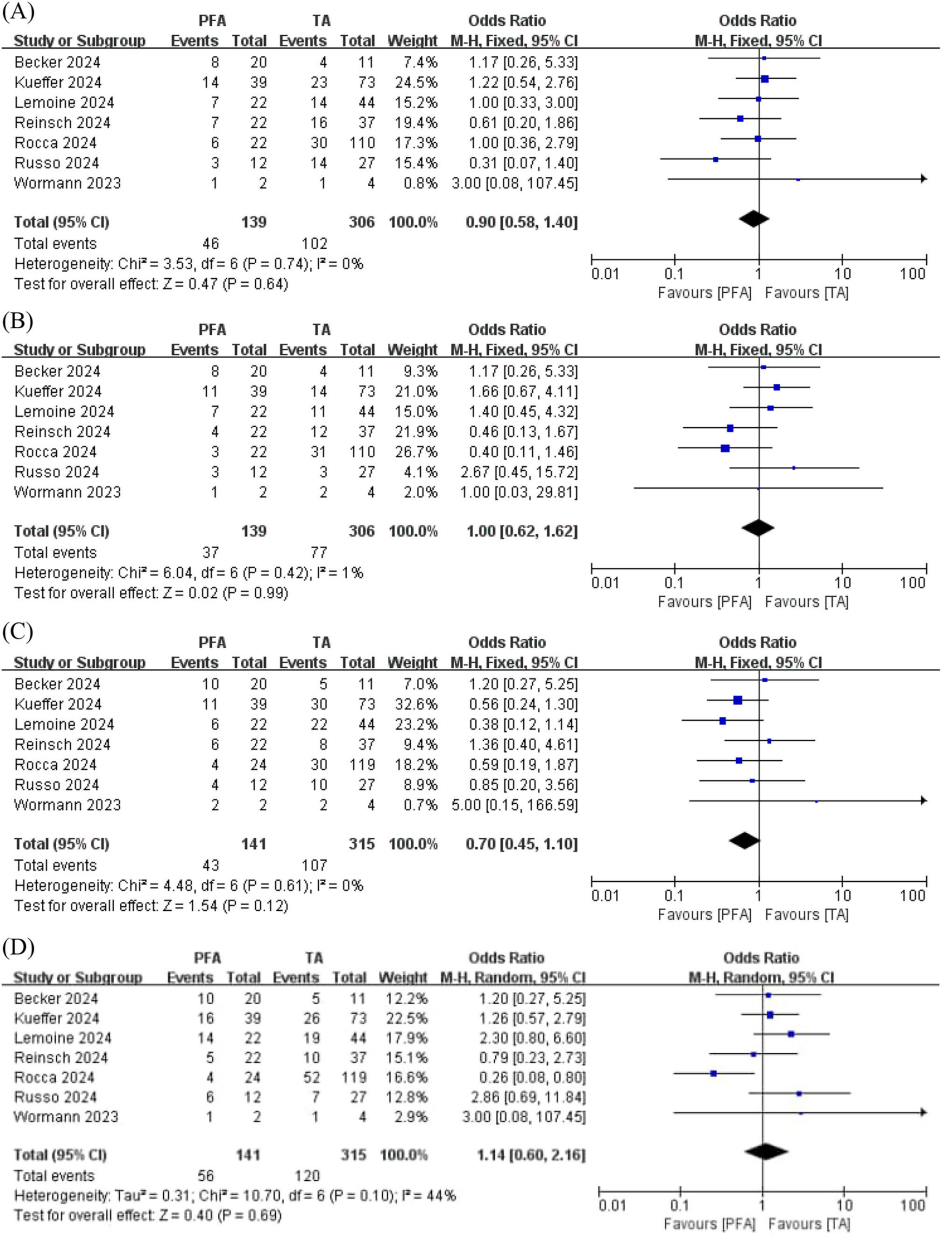
The forest plot displays the secondary endpoints when comparing PFA and TA for atrial fibrillation. (A) left superior PV, (B) left inferior PV, (C) right superior PV, (D) right inferior PV. CI, confidence interval; PFA, pulsed field ablation; PV, pulmonary vein; TA, thermal ablation.

After PFA, the RIPV (56/141; 39.7%) was the most frequently reconnected PV, followed by the LSPV (46/139; 33.1%), RSPV (43/141; 30.5%), and LIPV (37/139; 26.6%). After TA, the RIPV (120/315; 38.1%) remained the most frequently reconnected PV, followed by the RSPV (107/315; 34.0%), LSPV (102/306; 33.3%), and LIPV (77/306; 25.2%). No significant differences in reconnection rates were observed between the groups for LSPV (OR 0.90; 95% CI, 0.58–1.40; *p* = 0.64; I^2^ = 0%; Figure 3A), LIPV (OR 1.00; 95% CI, 0.62–1.62; *p* = 0.99; I^2^ = 1%; Figure 3B), RSPV (OR 0.70; 95% CI, 0.45–1.10; *p* = 0.12; I^2^ = 0%; Figure 3C), and RIPV (OR 1.14; 95% CI, 0.60–2.16; *p* = 0.69; I^2^ = 44%; Figure 3D).

### 3.4 Subgroup Analysis

The rates of repeat procedures were comparable between the PFA group (221/1986; 11.1%) and the CBA group (403/3103; 13.0%) (OR 0.75; 95% CI, 0.54–1.04; *p* = 0.09; I^2^ = 64%; Figure S1‐upper panel). Additionally, a separate subgroup analysis demonstrated similar results for the PFA group (174/1552; 11.2%) and the RFA group (233/1667; 14.0%) (OR 0.78; 95% CI, 0.59–1.03; *p* = 0.08; I^2^ = 31%; Figure S1‐lower panel).

PVI durability per patient was observed in 44% (44/100) of the PFA group and 32% (56/175) of the CBA group. However, this difference was not statistically significant (OR 1.35; 95% CI, 0.77–2.34; *p* = 0.29; I^2^ = 0%; Figure S2‐upper panel). Similarly, no statistically significant difference in PVI durability per patient was found in another subgroup analysis (PFA: 35.1% vs. RFA: 28.2%; OR 1.53; 95% CI, 0.88–2.69; *p* = 0.14; I^2^ = 0%; Figure S2‐lower panel).

There was no significant difference in the durability of PVI per vein between the PFA group (415/582; 71.3%) and the CBA group (614/903; 68.0%) (OR 1.02; 95% CI, 0.81–1.30; *p* = 0.85; I^2^ = 0%; Figure S3‐upper panel). In contrast, the PFA group (329/475; 69.2%) exhibited a higher rate of PVI durability per vein compared to the RFA group (528/830; 63.6%) (OR 1.32; 95% CI, 1.03–1.70; *p* = 0.03; I^2^ = 21%; Figure S3‐lower panel).

The reconnection rate of the LSPV did not reach statistical significance in either subgroup (PFA: 32.5% vs. CBA: 25.4%; OR 1.34; 95% CI, 0.72–2.49; *p* = 0.35; I^2^ = 0%) (PFA: 33.3% vs. RFA: 39.2%; OR 0.75; 95% CI, 0.45–1.25; *p* = 0.28; I^2^ = 0%) (Figure S4A).

The reconnection rate of the LIPV was the same in both the PFA group (21/83; 25.3%) and the CBA group (30/130; 23.1%) (OR 1.16; 95% CI, 0.48–2.80; *p* = 0.74; I^2^ = 38%), as well as between the PFA group (30/117; 25.6%) and the RFA group (47/176; 26.7%) (OR 0.88; 95% CI, 0.49–1.58; *p* = 0.67; I^2^ = 0%) (Figure S4B).

For the RSPV, no statistically significant differences were observed in either subgroup (PFA: 24.7% vs. CBA: 32.6%; OR 0.63; 95% CI, 0.34–1.19; *p* = 0.16; I^2^ = 0%) (PFA: 31.1% vs. RFA: 35.0%; OR 0.71; 95% CI, 0.42–1.19; *p* = 0.19; I^2^ = 0%) (Figure S4C).

No statistically significant differences were noted for the RIPV within either subgroup. (PFA: 40.0% vs. CBA: 40.7%; OR 0.98; 95% CI, 0.28–3.46; *p* = 0.97; I^2^ = 76%) (PFA: 35.3% vs. RFA: 36.1%; OR 0.93; 95% CI, 0.48–1.82; *p* = 0.84; I^2^ = 31%) (Figure S4D).

The comparative results of the two groups are summarized in Table 1 for convenient reference and comparison.

**TABLE 1:** Summary of comparison outcomes between PFA and TA.

Items	No. of studies	PFA (n)	TA (n)	OR (95% CI)	I^2^ (%)	P
**Pooled analysis**						
Re‐ablation rate	14	277/2597; 10.7%	636/4770; 13.3%	0.77 (0.59, 1.00)	59	0.05
PVI durability per patient	10	61/156; 39.1%	104/345; 30.1%	1.42 (0.92, 2.19)	0	0.12
PVI durability per vein	11	504/715; 70.5%	1142/1733; 65.9%	1.13 (0.93, 1.38)	0	0.22
Reconnection rate of LSPV	7	46/139; 33.1%	102/306; 33.3%	0.90 (0.58, 1.40)	0	0.64
Reconnection rate of LIPV	7	37/139; 26.6%	77/306; 25.2%	1.00 (0.62, 1.62)	1	0.99
Reconnection rate of RSPV	7	43/141; 30.5%	107/315; 34.0%	0.70 (0.45, 1.10)	0	0.12
Reconnection rate of RIPV	7	56/141; 39.7%	120/315; 38.1%	1.14 (0.60, 2.16)	44	0.69
**Subgroup analysis**						
**PFA versus CBA**						
Re‐ablation rate	10	221/1986; 11.1%	403/3103; 13.0%	0.75 (0.54, 1.04)	64	0.09
PVI durability per patient	6	44/100; 44.0%	56/175; 32.0%	1.35 (0.77, 2.34)	0	0.29
PVI durability per vein	8	415/582; 71.3%	614/903; 68.0%	1.02 (0.81, 1.30)	0	0.85
Reconnection rate of LSPV	3	27/83; 32.5%	33/130; 25.4%	1.34 (0.72, 2.49)	0	0.35
Reconnection rate of LIPV	3	21/83; 25.3%	30/130; 23.1%	1.16 (0.48, 2.80)	38	0.74
Reconnection rate of RSPV	3	21/85; 24.7%	44/135; 32.6%	0.63 (0.34, 1.19)	0	0.16
Reconnection rate of RIPV	3	34/85; 40.0%	55/135; 40.7%	0.98 (0.28, 3.46)	76	0.97
**PFA versus RFA**						
Re‐ablation rate	9	174/1552; 11.2%	233/1667; 14.0%	0.78 (0.59, 1.03)	31	0.08
PVI durability per patient	7	34/97; 35.1%	48/170; 28.2%	1.53 (0.88, 2.69)	0	0.14
PVI durability per vein	7	329/475; 69.2%	528/830; 63.6%	1.32 (1.03, 1.70)	21	0.03
Reconnection rate of LSPV	6	39/117; 33.3%	69/176; 39.2%	0.75 (0.45, 1.25)	0	0.28
Reconnection rate of LIPV	6	30/117; 25.6%	47/176; 26.7%	0.88 (0.49, 1.58)	0	0.67
Reconnection rate of RSPV	6	37/119; 31.1%	63/180; 35.0%	0.71 (0.42, 1.19)	0	0.19
Reconnection rate of RIPV	6	42/119; 35.3%	65/180; 36.1%	0.93 (0.48, 1.82)	31	0.84

Abbreviations: CBA, cryoballoon ablation; CI, confidence interval; LIPV, left inferior pulmonary vein; LSPV, left superior pulmonary vein; OR, odds ratio; PFA, pulsed field ablation; PVI, pulmonary vein isolation; RFA, radiofrequency ablation; RIPV, right inferior pulmonary vein; RSPV, right superior pulmonary vein; TA, thermal ablation.

### 3.5 Sensitivity Analysis

The outcomes of the rate of repeat procedures (59%) and the incidence of RIPV reconnection (44%) exhibited moderate heterogeneity, with I^2^ > 25% and < 75%. The pooled analysis of the aforementioned outcomes was conducted utilizing a random‐effects model to mitigate the potential impacts of inter‐study heterogeneity. Sequential exclusion analysis did not alter the degree of heterogeneity in repeat procedure rates. Consequently, the sensitivity analysis of the sequential exclusions corroborates the robustness of this finding.

We identified the study conducted by Rocca et al. [28] as the primary source of heterogeneity in the incidence of RIPV reconnection. After excluding this study, no evidence of heterogeneity was observed (I^2^ = 0%), and the lack of statistical significance between groups remained unchanged (Figure S5). The heterogeneity may be attributed to the significantly lower frequency of RIPV reconnection among PFA patients in the study by Rocca et al. (*p* = 0.04) [28].

### 3.6 Publication Bias

The funnel plots did not reveal any evidence of publication bias for the selected outcomes of durability of PVI both per patient and per vein (Figure S6).

## 4 Discussion

### 4.1 Main Findings

To the best of our knowledge, this study represents the first meta‐analysis comparing outcomes repeat ablation due to recurrent ATa after a first PFA and TA. The principal findings can be summarized as follows:

1. PFA demonstrated a trend toward a decreased incidence of repeat procedures in comparison to TA.
2. Upon repeat ablation, the durability of PVI, assessed both per patient and per vein, was found to be comparable between PFA and TA. Subgroup analyses demonstrated a significant improvement in PVI durability on a per‐vein level in patients treated with PFA compared to those initially treated with RFA.
3. The RIPV was the most frequently reconnected PV in both PFA and TA. There were no differences observed in the reconnection rates of individual PVs between the two groups.

It is critical to acknowledge that patients undergoing repeat ablation in clinical practice represent a negatively selected population with inherently higher risks of PV reconnection, compared to those systematically remapped in clinical studies. This selection bias may artificially inflate the observed PV reconnection rates in our analysis, as repeat mapping in the included studies was predominantly conducted only in cases of clinical recurrence for both groups. Consequently, the reported PVI durability estimates may not reflect the true success rate in the broader ablation population, particularly among asymptomatic patients. Moreover, 277/2597 PFA patients and 636/4770 TA patients underwent repeat ablation, but the assessments of PVI durability involved only 156 PFA patients and 345 TA patients (the significant reduction in sample size is due to the absence of PVI durability data in the four original studies). The limited sample size may obscure the genuine difference between PFA and TA. Future trials mandating protocol‐driven remapping regardless of symptoms are needed to mitigate this bias.

Our analysis indicated that PFA demonstrated a trend toward a lower incidence of repeat procedures relative to TA. Repeat ablation may be more prevalent among patients with a history of coronary artery bypass grafting, a common left PV orifice, non‐paroxysmal AF, arterial hypertension, and advanced age. This is likely due to the higher prevalence of triggers within this patient population [31]. Given the absence of baseline characteristics for patients undergoing repeat ablation, it is not possible to identify predictors of repeat ablation within the scope of this study.

PVI durability is the most reliable predictor of freedom from AF recurrence and consequently holds paramount importance in the interventional treatment of AF [32]. For repeat ablation after TA, multicenter RCTs have reported durability rates 10%–30% at the patient level and 46%–64% at the vein level [33, 34], in contrast to rates of 50% at the patient level and 80% at the vein level observed in repeat procedures for all‐comers [35]. Furthermore, multiple observational studies assessing repeat procedures in patients experiencing symptomatic recurrences have demonstrated superior PVI durability for CBA compared to RFA (Table S1). Conversely, the use of the novel non‐thermal PFA device in a premarket study, where all patients underwent mandatory remapping procedures, showed a marked improvement in PVI durability, reaching 84% of patients and 96% of veins [36]. This data indicates that PFA technology is capable of achieving transmural and durable PVI in the majority of patients, thereby significantly enhancing prognosis following AF ablation. However, postmarket durability data after index PFA versus TA in patients experiencing clinical recurrences remain limited.

In our study, the durability of ablation per patient and per vein was found to be nearly identical between PFA and CBA. Our findings do not support the initial optimism that PFA integrated into a pentaspline catheter would significantly enhance PVI durability (96%) [36] compared to CBA (80%) [37], as previously reported in two independent studies that included mandatory remapping studies. The latter study also revealed that in patients with paroxysmal AF (PAF) who underwent second‐generation CBA, a reduced number of reconnection gaps were observed [37]. This improvement underscores the ongoing advancements in TA techniques. Similarly, as PFA devices continue to evolve, they may also contribute to enhanced PVI durability. Enhancing operator experience along with technical advancements in the second‐generation pentaspline PFA catheter may potentially lead to a further reduction in PV reconnection. To draw more definitive conclusions, larger multicenter head‐to‐head comparisons between the two single‐shot devices are essential.

Regarding RFA, our study demonstrated a substantially higher incidence of PV reconnection when compared to PFA. Although RFA exhibited a higher reconnection rate, no significant difference was observed in the effectiveness of arrhythmia control between treatment groups in clinical practice [8]. This indicates that factors beyond the technical approach itself may significantly influence treatment outcomes. Furthermore, studies utilizing pharmacological induction protocols to systematically map and ablate triggers initiating AF have demonstrated that other sources of ectopic beats can be elicited at a higher frequency in non‐PAF patients. However, these sources still occur in 20%–30% of PAF patients, depending on the specific induction protocol and criteria employed for their identification [38]. Left atrial substrate abnormalities, including low voltage areas, have been documented in approximately 10% of cases [39]. These observations may explain a significant proportion of recurrences due to atrial ectopies originating from extra‐PV sites and abnormal atrial substrates, which can contribute to arrhythmia recurrence and maintenance independently of durable PVI. In conclusion, we posit that establishing whether PFA confers any additional benefits for arrhythmia control will necessitate larger‐scale studies and an arrhythmia monitoring strategy employing implantable loop recorders to more comprehensively elucidate differences in AF control and residual burden.

The relatively high incidence of reconnections in the RIPV during CBA and PFA, as compared to other PVs, is likely attributable to anatomical challenges in positioning the ablation devices. Both the cryothermy and pentaspline catheters necessitate the use of a larger, less flexible sheath, which complicates precise placement. This challenge is particularly pronounced if the transseptal puncture is performed too high or too posterior relative to the RIPV, or if the RIPV is located too low relative to an optimal transseptal puncture site [34]. Given that there are no indications of extracardiac injuries such as phrenic nerve palsy or significant esophageal damage associated with PFA, additional applications of PFA can enhance efficacy without compromising safety. Precise placement or repositioning of the catheter and applying additional treatments at the inferior‐anterior aspect of the RIPV may be an effective strategy to improve the durability of PVI.

The presence of left common PV (LCPV) has previously been associated with elevated reconnection rates for CBA [40], which may potentially favor the utilization of alternative energy modalities in this anatomical context. PFA using the pentaspline catheter may be limited in overcoming this obstacle due to its single‐shot design, which restricts adaptability to anatomical variations. In contrast, only two of the included studies reported LCPV reconnection rates in our analysis [25, 28]. Specifically, LCPV was reconnected in 4 out of 8 (50%) repeat procedures following PFA, compared to 5 out of 12 (42%) after CBA. Additional and more comprehensive data are required to determine which device may be more effective in treating LCPVs.

### 4.2 Comparisons to Previous Meta‐Analysis

PVI durability in protocol‐based and planned remapping studies were comparable after adjustment for patient characteristics in a recent meta‐analysis: PVI durability of at least one PV (per patient analysis) were 70% for PFA, 62% for laser, 54% for CBA, and 46% for RFA, while a per vein analysis revealed durability of 87% for PFA, 84% for laser, 79% for CBA, and 71% for RFA [41]. However, it is important to note that the study was based on a single‐arm meta‐analysis, and the PFA data were derived from only two pre‐commercial trials (both published in 2021) [36, 42]. Conversely, the primary objective of our meta‐analysis was to compare the durability of PVI achieved through PFA versus two classic thermal energy sources, namely CBA and RFA. Furthermore, repeat mapping was primarily performed for clinical recurrence, and not mandated by protocol for the individual studies included in the meta‐analysis. Lastly, our analysis included studies published up to January 2025, ensuring that our findings are consistent with the latest advancements in the field.

## 5 Clinical Implications

PFA demonstrates potential for lasting PVI. Despite being introduced fewer than 4 years ago, the pentaspline PFA demonstrates a reconnection rate comparable to that of the CBA system, which has been in use for over 15 years. Moreover, it exhibits a superior reconnection rate compared to the RFA system, which has been available for more than two decades and has undergone multiple iterations in catheter design. Experienced operators who had prior exposure to CBA demonstrated superior outcomes in terms of PVI durability, underscoring the significant learning curve associated with over‐the‐wire PVI ablation [43]. Operators without previous experience may encounter a more prolonged learning phase, and comprehensive training is essential for achieving proficiency. Accurate catheter positioning is critical to prevent reconnections; specific attention should be given to the the inferior portion of the RIPV. The introduction of second‐generation PFA catheters integrated with 3D‐EAM promises enhanced catheter placement and improved lesion assessment, potentially leading to better PVI durability. Moreover, the deployment of supplementary applications could constitute an efficacious strategy to augment the durability of PVI. However, further research is imperative to ascertain whether enhanced PFA applications can reduce reconnections without compromising safety.

## 6 Limitations

First and foremost, only a subset of patients with symptomatic recurrent ATa following PFA or TA‐guided PVI have been investigated. Consequently, the durability of PVI in the entire patient population remains uncertain. Secondly, the pooled analysis should be interpreted with caution, as only one of the 14 eligible trials utilized a randomized design. Nevertheless, it is noteworthy that 85% of the included studies were rated as high quality. This composition reflects real‐world experiences and offers generalizability advantages for contemporary practice. Thirdly, the demographic baseline characteristics of patients undergoing re‐ablation were unavailable, precluding the identification of predictors for PVI durability. Similarly, extra‐PV ablation is seldom performed during the initial PVI, limiting our ability to evaluate its durability during subsequent re‐ablation procedures. Additional data are required to adequately assess the durability of extra‐PV lesions. Fourthly, all included studies utilized the FARAPULSE™ system exclusively, and thus their findings cannot be extrapolated to other PFA systems currently available in the market. Each PFA system employs unique waveforms and energy settings, which may lead to varying outcomes. Ultimately, the original study included in the analysis did not consistently perform repeat ablation for all patients with a documented history of recurrent ATa, and the criteria for determining the necessity of reablation varied both within and between participating centers, potentially introducing selection bias.

## 7 Conclusion

At the time of repeat ablation following initial PVI using PFA or TA, PFA exhibited a trend toward a reduced incidence of repeat procedures compared to TA. The lesion durability and reconduction rates in individual PVs were found to be comparable, with the exception that PFA exhibited superior durable isolation across all veins compared to RFA. The RIPV is the most frequently reconnected PV.

## Author Contributions

Concept/design: Yin Zhang. Data analysis/interpretation: Jin‐Cheng Chen, Yin‐Jun Mao. Drafting article: Jin‐Cheng Chen, Yin‐Jun Mao. Critical revision of article: All. Approval of the final version of the manuscript: All.

## Ethics Statement

The authors have nothing to report.

## Conflicts of Interest

The authors declare no conflicts of interest.

## Supporting information

Supplementary materials.

## Data Availability

All data generated or analyzed during this study are included in this article (and its supporting material files).
