# Supplementary material for: Meta‐Analysis of Thermal Versus Pulse Field Ablation for Pulmonary Vein Isolation Durability in Atrial Fibrillation: Insights From Repeat Ablation
Source: Clin Cardiol. 2025 May 28;48(6):e70151. doi: 10.1002/clc.70151 (PMC12120194; doi:10.1002/clc.70151)
Supplement: Supplementary file 1 — Supplementary materials. [file CLC-48-e70151-s001.docx]

**Table S1** Literature overview of trials comparing durability between cryoablation (CBA) and radiofrequency ablation (RFA) for pulmonary vein isolation (PVI)

| **Study** | **Year** | **Patient** | **PVI durability % per patient** | | **PVI durability % per vein** | |
| --- | --- | --- | --- | --- | --- | --- |
|  |  | **CBA/RFA** | **CBA** | **RFA** | **CBA** | **RFA** |
| Aryana^1^ | 2015 | 186/174 | 52.7 | 39.1 | 81.2 | 65.4 |
| Ciconte^2^ | 2016 | 26/30 | 30.8 | 20.0 | 79.6 | 63.9 |
| Buist^3^ | 2018 | 20/33 | 15.0 | 3.0 | 63.2 | 41.9 |
| Kuck^4^ | 2019 | 36/53 | 21.9 | 17.3 | 63.7 | 46.2 |
| Cheung^5^ | 2020 | 16/36 | 8.3 | 12.5 | 56.8 | 53.6 |
| Obergassel^6^ | 2024 | 247/363 | 18 | 15 | 52 | 44 |

**Supplementary References:**

1. Aryana A, Singh SM, Kowalski M, et al. Acute and Long-Term Outcomes of Catheter Ablation of Atrial Fibrillation Using the Second-Generation Cryoballoon versus Open-Irrigated Radiofrequency: A Multicenter Experience. J Cardiovasc Electrophysiol. 2015;26(8):832-9.

2. Ciconte G, Velagic V, Mugnai G, et al. Electrophysiological findings following pulmonary vein isolation using radiofrequency catheter guided by contact-force and second-generation cryoballoon: lessons from repeat ablation procedures. EUROPACE. 2016;18(1):71-7.

3. Buist TJ, Adiyaman A, Smit J, Ramdat MA, Elvan A. Correction to: Arrhythmia-free survival and pulmonary vein reconnection patterns after second-generation cryoballoon and contact-force radiofrequency pulmonary vein isolation. CLIN RES CARDIOL. 2018;107(6):530.

4. Kuck KH, Albenque JP, Chun KJ, et al. Repeat Ablation for Atrial Fibrillation Recurrence Post Cryoballoon or Radiofrequency Ablation in the FIRE AND ICE Trial. Circ Arrhythm Electrophysiol. 2019;12(6):e7247.

5. Cheung CC, Deyell MW, Macle L, et al. Repeat Atrial Fibrillation Ablation Procedures in the CIRCA-DOSE Study. Circ Arrhythm Electrophysiol. 2020;13(9):e8480.

6. Obergassel J, Nies M, Taraba S, et al. Pulmonary vein reconnection and repeat ablation characteristics following cryoballoon-compared to radiofrequency-based pulmonary vein isolation. J Cardiovasc Electrophysiol. 2024;35(9):1766-78.

**Table S2** Patient demographics

| **Study,**  **year** | **PFA vs TA** | **Patients,**  **n** | **Age,**  **years** | **Male,**  **%** | **PAF,**  **%** | **BMI,**  **kg/m2** | **HTN,**  **%** | **DM,**  **%** | **Stroke/**  **TIA, %** | **CHA_2_DS-**  **VASc** | **CAD,**  **%** | **CHF,**  **%** | **DPA,**  **%** | **LVEF,**  **%** | **LAD,**  **mm** |
| --- | --- | --- | --- | --- | --- | --- | --- | --- | --- | --- | --- | --- | --- | --- | --- |
| Badertsche 2023 | PFA/CBA | 106/75 | 65/64 | 63/64 | 61/68 | 27±3.7/  27±3.7 | 60/47 | 11/8 | NR | NR | 9/8 | NR | 43/27 | 57±8.6/  58±9.1 | 41±6.4/  40±6.3 |
| Reddy  2023 | PFA/CBA/RFA | 305/135/167 | 62/62/63 | 66/62/67 | 100/100/100 | 28.3±4.6/  29.6±4.8/  28.5±4.8 | 57/48/56 | 10.8/9.6/11 | 3.9/3.7/6 | 1.7±1.2/  1.6±1.3/  1.8±1.2 | 10.5/16.3/17.4 | 19.3/20.7/18.6 | 43.6/50.4/  43.7 | NR | 38.8±5.7/  40.0±5.7/  39.3±5.9 |
| Urbanek  2023 | PFA/CBA | 200/200 | 71/68 | 59/54 | 58/63.5 | 27±5.2/  27±4.4 | 66/71 | 14/16.5 | 5/7.5 | 2±1.5/  3±2.2 | 14/13.5 | 13.5/11.5 | NR | NR | 41±6.7/  40±5.9 |
| Wörmann  2023 | PFA/RFA | 57/57 | 67/67 | 33/40 | 3030 | 28±5/  27±4 | 65/60 | 16/14 | NR | 3/3 | 25/19 | NR | NR | 56±6/  56±9 | 40±6/  38±4 |
| Becker  2024 | PFA/RFA | 161/161 | 65/65 | 64/63 | 78/76 | 26±3.7/  27±3.0 | 50/48 | 9/9 | NR | 2±1.5/  2±1.5 | NR | 17/17 | NR | NR | 41±6/  40±5 |
| Calvert  2024 | PFA/CBA/RFA | 208/325/174 | 64/64/63 | 65/64/67 | 56/59/62 | 29±5.2/  29±4.4/  29±4.4 | 41/46/50 | 7/8/8 | 7/5/5 | 2±1.5/  2±1.5/  2±1.5 | 10/11/7 | 17/13/17 | NR | NR | NR |
| Chaumont  2024 | PFA/CBA | 151/150 | 61/63 | 62/62 | 74/73 | 27±4.6/  27±4.2 | 38/41 | 9/7 | 3/7 | 1±0.4/  2±0.7 | 6/4 | 5/2 | NR | NR | NR |
| Kueffer  2024 | PFA/CBA/RFA | 214/190/129 | 69/68/68 | 77/73/70 | 0/0/0 | 28±5.5/  29±5.6/  29±5.2 | 61/68/60 | 18/11/19 | 6/11/7 | 3±1.5/  3±1.5/  3±2.2 | 21/17/19 | NR | NR | 55±11.1/  55±11.1/  55±11.5 | NR |
| Lemoine  2024 | PFA/CBA | 191/359 | 67/67 | 66/62 | 51/35 | 26±4.4/  27±5.2 | 68/67 | 12/9 | 7/9 | 2.7±1.7/  2.7±1.6 | 26/19 | 31/35 | NR | NR | NR |
| Maurhofer  2024 | PFA/CBA/RFA | 40/80/80 | 63/62/62 | 75/73/79 | 100/100/100 | 25.9±3.9/  26.2±3.4/  25.6±3.6 | 65/63/60 | 8/10/11 | 5/5/10 | NR | 20/18/13 | NR | NR | 60±3.7/  60±6.8/  60±3.7 | 42±5.8/  42±5.2/  41±7.1 |
| Reinsch  2024 | PFA/RFA | 201/209 | 68/68 | 56/53 | 100/100 | 27±4/  27±4 | 66/67 | 11/13 | 5/11 | NR | 9/10 | NR | NR | NR | NR |
| Rocca  2024 | PFA/CBA/RFA | 174/655/743 | 62/61/64 | 63/60/52 | 100/100/100 | 27±4.8/  27±4.6/  26±6.7 | 45/43/42 | 9/7/10 | 5/5/4 | 2±1.5/  2±1.5/  2±1.5 | 6/7/6 | 8/9/8 | NR | 59±4.2/  58±7.5/  58±6.9 | NR |
| Russo  2024 | PFA/RFA | 192/342 | 65/63 | 63/65 | 68/70 | 24.7±1.1/  24.7±1.1 | 63/60 | 29/12 | NR | 2±1.5/  2±0.7 | 14/16 | 27/23 | 34/37 | 60±5.2/  60±3.7 | NR |
| Van  2024 | PFA/CBA | 473/1241 | 65/64 | 64/69 | 61/66 | 26.8±3.7/  26.8±5.1 | NR | 9/7 | NR | NR | NR | NR | NR | 55±5/  55±6 | NR |

BMI: body-mass index; CAD: coronary artery disease; CBA: cryoballoon ablation; CHF: congestive heart failure; DM: diabetes mellitus; DPA: dyslipidemia; HTN: hypertension; LAD: left atrial diameter; LVEF: left ventricular ejection fraction; NR: not reported; PAF: paroxysmal atrial fibrillation; PFA: pulsed-field ablation; RFA: radiofrequency ablation; TA: thermal ablation; TIA: transient ischemic attack

**Table S3** Study characteristics

| **Study** | **Badertscher**  **2023** | **Reddy**  **2023** | **Urbanek**  **2023** | **Wörmann**  **2023** | **Becker**  **2024** | **Calvert**  **2024** | **Chaumont**  **2024** | **Kueffer**  **2024** | **Lemoine**  **2024** | **Maurhofer**  **2024** | **Reinsch**  **2024** | Rocca  2024 | **Russo**  **2024** | **Van**  **2024** |
| --- | --- | --- | --- | --- | --- | --- | --- | --- | --- | --- | --- | --- | --- | --- |
| Study  design | Prospective  Single center | RCT  Multi-center | Retrospective  Single center | Retrospective  Single center | Retrospective  Multi-centerr | Retrospective  Single center | Prospective  Single center | Prospective  Single center | Prospective  Single center | Prospective  Single center | Retrospective  Single center | Retrospective  Multi-center | Retrospective  Multi-center | Retrospective  Single center |
| Location | Switzerland | USA | Germany | Germany | Belgium | UK | France | Switzerland | Germany | Switzerland | Germany | USA; Italy | Italy | Netherlands |
| Study population | Patients with PAF or persAF that underwent first-time PVI using PFA or CBA | Patients with symptomatic PAF resistant to AAD,  LVEF>40% and LAD<5.5 cm | Patients with symptomatic AF, who underwent CBA or PFA were analyzed | Patients undergoing de novo PFA or HPSD for symptomatic PAF or persAF were included | Patients with symptomatic PAF or persAF, all scheduled for their initial PVI procedure. | Patients who underwent first-time AF CA were included | Patients who underwent a first PVI using either PFA or CBA | Patients undergoing a first PVI for persAF were included | All consecutive patients undergoing repeat ablation after initial PFA or CBA for AF. | Patients with  PAF undergoing first catheter ablation | Patients with symptomatic PAF, who underwent HPSD or PFA were analyzed | Patients undergoing ablation‐based first time PVI were analyzed | Patients of at least 18 years of age, with symptomatic PAF or persAF undergoing a first AF CA using PFA or HPSD at there centers. | Eligible participants were adults aged  ≥18 years who had undergone either CBA or  PFA ablation as their initial AF treatment. |
| PFA  waveform | Faraware (Boston  Scientifc)  Biphasic | Farawave  (Boston  Scientifc)  Biphasic | Farawave  ( Boston  Scientifc)  Biphasic | Farawave (Boston Scientifc)  NR | Farawave (Boston Scientifc)  Biphasic | Farawave  (Boston  Scientifc)  Biphasic | Farawave  (Boston Scientifc)  NR | Farawave  (Boston Scientifc)  Biphasic | Farawave (Boston  Scientifc)  NR | Farawave  (Boston Scientifc)  Biphasic | Farawave (Boston Scientifc)  NR | Farawave  (Boston Scientifc)  Monophasic; Biphasic | Farawave  (Boston  Scientifc)  Biphasic | Farawave  (Boston Scientifc)  NR |
| CBA | 28-mm CB catheter (Boston Scientific) | 23-mm or 28-mm CB catheter (2nd  generation,  Arctic Front  Advance, Medtronic) | 28 mm CB catheter  (2nd generation, Arctic Front Advance, Medtronic) | None | None | 28-mm CB catheter (Arctic Front Advance,  Medtronic) | 28-mm CB catheter (Arctic Front Advance,  Medtronic) | 28-mm CB catheter (Arctic Front Advance,  Medtronic) | 28-mm CB catheter (Arctic Front Advance,  Medtronic) | 28-mm CB catheter  (Arctic Front Advance,  Medtronic | None | 23-mm or 28-mm CB catheter (2nd  generation,  Arctic Front  Advance, Medtronic); 28-mm CB catheter (Boston Scientific) | None | 27-mm CB catheter (Arctic Front,  Medtronic) |
| RFA | None | None | Irrigated, CF (Abbott; Biosense Webster) | Irrigated (Abbott) | Irrigated, CF (Biosense Webster) | Irrigated, CF (Biosense Webster) | None | Irrigated, CF (Biosense Webster) | None | None | Irrigated (Biosense Webster) | Irrigated, CF (Biosense Webster) | Irrigated, CF (Biosense Webster) | None |
| Monitoring | 12-lead ECG and 7-day Holter were performed at 3, 6, and 12 months. 12-lead ECG or Holter ECG if  symptomatic | 72-h Holter at 6 and 12 months, and trans-telephonic ECG weekly  for symptoms | 72-h Holter at 6 and 12 months | Follow‐up consisted of out‐clinic patients' visits at 3 and 12 months after PVI, photopletysmogram app‐based tele‐consultation, 48 h Holter ECG and CIED interrogation if applicable. | Physical exam and ECG were done at 1, 3, 6 months post-procedure. A 24h Holter was done at 3 months, and a 2-6 days Holter at 6 months post-procedure or if symptoms occurred. | Outpatient clinic follow-up usually occurs at 3, 6, and 12 months with 12-lead ECG performed at the  appointment. If AF symptoms occur, ambulatory ECG monitoring was arranged. | The 24-hour Holter monitoring was performed at 3- month follow-up in addition to a systematic 4-month and 1-year follow-up visit. Center also made systematic phone contact after 1-year follow-up | Follow-up visits including a 7-day-Holter  ECG were scheduled at 3, 6, and 12 months after PVI. | None | 7-day Holter at 3, 6, and 12 months | The patients were scheduled for clinical and 5-day Holter ECG follow-up at 3, 6 and 12 months | Follow-up visits were scheduled at 1, 3, 6 months, then every 6 months. Each visit included physical exam, 12-lead ECG, and 24h Holter monitoring. If arrhythmia recurrence symptoms occurred, 7-day Holter monitoring was prescribed. | At 3 months after CA and every 6 months thereafter, 24h Holter monitoring, 12-lead ECG, and clinical controls were scheduled. If symptoms were reported, additional 12-lead ECG or Holter monitoring was prescribed. | None |
| Follow-up | 12 months | 12 months | 12 months | 12 months | 6 months | 12 months | 12 months | 12 months | None | 12 months | 12 months | 6 months | 6 months | 6 months |

AAD: Antiarrhythmic drug; AF: atrial fibrillation; CA: Catheter ablation; CBA: cryoballoon ablation; CF: contact force; CMR: cardiac magnetic resonance; HPSD: high‐power short‐duration ablation; LAD: left atrial diameter; LVEF: left ventricular ejection fraction; NR: not reported; PAF: paroxysmal atrial fibrillation; persAF: persistent atrial fibrillation; PFA: pulsed field ablation; PVI: pulmonary vein isolation; RCT: randomised controlled trial; RFA: radiofrequency ablation

**Table S4** The Cochrane Bias Risk Assessment Tool assessment results of randomized controlled trial

| **Author name: Reddy (2023)** | |
| --- | --- |
| **Items** | **Response options** |
| *Random sequence generation (selection bias)* | **Low risk** |
| *Allocation concealment (selection bias)* | **Low risk** |
| *Blinding of participants and personnel (performance bias)* | **Low risk** |
| *Blinding of outcome assessment (detection bias)* | **Low risk** |
| *Incomplete outcome data (attrition bias)* | **Low risk** |
| *Selective reporting (reporting bias)* | **Low risk** |
| *Other bias* | **Low risk** |

**Table S5** Newcastle-Ottawa Scale assessment results of cohort studies

| **Author name:** **Badertscher (2023)** | |
| --- | --- |
| **Items** | **Response options** |
| Selection | |
| *Representativeness of the exposed cohort* | *** |
| *Selection of the non exposed cohort* | *** |
| *Ascertainment of exposure* | *** |
| *Demonstration that outcome of interest was not present at start of study* | *** |
| Comparability | |
| *Comparability of cohorts on the basis of the design or analysis* | **** |
| Outcome | |
| *Assessment of outcome* | *** |
| *Was follow-up long enough for outcomes to occur* | *** |
| *Adequacy of follow up of cohorts* | *** |

| **Author name:** **Urbanek (2023)** | |
| --- | --- |
| **Items** | **Response options** |
| Selection | |
| *Representativeness of the exposed cohort* | *** |
| *Selection of the non exposed cohort* | *** |
| *Ascertainment of exposure* | *** |
| *Demonstration that outcome of interest was not present at start of study* | *** |
| Comparability | |
| *Comparability of cohorts on the basis of the design or analysis* | **** |
| Outcome | |
| *Assessment of outcome* | *** |
| *Was follow-up long enough for outcomes to occur* | *** |
| *Adequacy of follow up of cohorts* | *** |

| **Author name: Wörmann (2023)** | |
| --- | --- |
| **Items** | **Response options** |
| Selection | |
| *Representativeness of the exposed cohort* | *** |
| *Selection of the non exposed cohort* | *** |
| *Ascertainment of exposure* | *** |
| *Demonstration that outcome of interest was not present at start of study* | *** |
| Comparability | |
| *Comparability of cohorts on the basis of the design or analysis* | ** |
| Outcome | |
| *Assessment of outcome* | *** |
| *Was follow-up long enough for outcomes to occur* | *** |
| *Adequacy of follow up of cohorts* | *** |

| **Author name: Becker (2024)** | |
| --- | --- |
| **Items** | **Response options** |
| Selection | |
| *Representativeness of the exposed cohort* | *** |
| *Selection of the non exposed cohort* |  |
| *Ascertainment of exposure* | *** |
| *Demonstration that outcome of interest was not present at start of study* | *** |
| Comparability | |
| *Comparability of cohorts on the basis of the design or analysis* | * |
| Outcome | |
| *Assessment of outcome* | *** |
| *Was follow-up long enough for outcomes to occur* | *** |
| *Adequacy of follow up of cohorts* | *** |

| **Author name: Calvert (2024)** | |
| --- | --- |
| **Items** | **Response options** |
| Selection | |
| *Representativeness of the exposed cohort* | *** |
| *Selection of the non exposed cohort* | *** |
| *Ascertainment of exposure* | *** |
| *Demonstration that outcome of interest was not present at start of study* | *** |
| Comparability | |
| *Comparability of cohorts on the basis of the design or analysis* | *** |
| Outcome | |
| *Assessment of outcome* | *** |
| *Was follow-up long enough for outcomes to occur* | *** |
| *Adequacy of follow up of cohorts* | *** |

| **Author name: Chaumont (2024)** | |
| --- | --- |
| **Items** | **Response options** |
| Selection | |
| *Representativeness of the exposed cohort* | *** |
| *Selection of the non exposed cohort* | *** |
| *Ascertainment of exposure* | *** |
| *Demonstration that outcome of interest was not present at start of study* | *** |
| Comparability | |
| *Comparability of cohorts on the basis of the design or analysis* | **** |
| Outcome | |
| *Assessment of outcome* | *** |
| *Was follow-up long enough for outcomes to occur* | *** |
| *Adequacy of follow up of cohorts* | *** |

| **Author name: Kueffer (2024)** | |
| --- | --- |
| **Items** | **Response options** |
| Selection | |
| *Representativeness of the exposed cohort* | *** |
| *Selection of the non exposed cohort* |  |
| *Ascertainment of exposure* | *** |
| *Demonstration that outcome of interest was not present at start of study* | *** |
| Comparability | |
| *Comparability of cohorts on the basis of the design or analysis* | *** |
| Outcome | |
| *Assessment of outcome* | *** |
| *Was follow-up long enough for outcomes to occur* | * |
| *Adequacy of follow up of cohorts* | *** |

| **Author name: Lemoine (2024)** | |
| --- | --- |
| **Items** | **Response options** |
| Selection | |
| *Representativeness of the exposed cohort* | *** |
| *Selection of the non exposed cohort* | * |
| *Ascertainment of exposure* | *** |
| *Demonstration that outcome of interest was not present at start of study* | *** |
| Comparability | |
| *Comparability of cohorts on the basis of the design or analysis* |  |
| Outcome | |
| *Assessment of outcome* | *** |
| *Was follow-up long enough for outcomes to occur* |  |
| *Adequacy of follow up of cohorts* | *** |

| **Author name: Maurhofer (2024)** | |
| --- | --- |
| **Items** | **Response options** |
| Selection | |
| *Representativeness of the exposed cohort* | *** |
| *Selection of the non exposed cohort* | * |
| *Ascertainment of exposure* | *** |
| *Demonstration that outcome of interest was not present at start of study* | *** |
| Comparability | |
| *Comparability of cohorts on the basis of the design or analysis* | **** |
| Outcome | |
| *Assessment of outcome* | *** |
| *Was follow-up long enough for outcomes to occur* | *** |
| *Adequacy of follow up of cohorts* | *** |

| **Author name: Reinsch (2024)** | |
| --- | --- |
| **Items** | **Response options** |
| Selection | |
| *Representativeness of the exposed cohort* | *** |
| *Selection of the non exposed cohort* | *** |
| *Ascertainment of exposure* | *** |
| *Demonstration that outcome of interest was not present at start of study* | *** |
| Comparability | |
| *Comparability of cohorts on the basis of the design or analysis* | *** |
| Outcome | |
| *Assessment of outcome* | *** |
| *Was follow-up long enough for outcomes to occur* | *** |
| *Adequacy of follow up of cohorts* | *** |

| **Author name: Rocca (2024)** | |
| --- | --- |
| **Items** | **Response options** |
| Selection | |
| *Representativeness of the exposed cohort* | *** |
| *Selection of the non exposed cohort* | *** |
| *Ascertainment of exposure* | *** |
| *Demonstration that outcome of interest was not present at start of study* | *** |
| Comparability | |
| *Comparability of cohorts on the basis of the design or analysis* | **** |
| Outcome | |
| *Assessment of outcome* | *** |
| *Was follow-up long enough for outcomes to occur* |  |
| *Adequacy of follow up of cohorts* | *** |

| **Author name: Russo (2024)** | |
| --- | --- |
| **Items** | **Response options** |
| Selection | |
| *Representativeness of the exposed cohort* | *** |
| *Selection of the non exposed cohort* | *** |
| *Ascertainment of exposure* | *** |
| *Demonstration that outcome of interest was not present at start of study* | *** |
| Comparability | |
| *Comparability of cohorts on the basis of the design or analysis* | **** |
| Outcome | |
| *Assessment of outcome* | *** |
| *Was follow-up long enough for outcomes to occur* |  |
| *Adequacy of follow up of cohorts* | *** |

| **Author name: Van (2024)** | |
| --- | --- |
| **Items** | **Response options** |
| Selection | |
| *Representativeness of the exposed cohort* | *** |
| *Selection of the non exposed cohort* | *** |
| *Ascertainment of exposure* | *** |
| *Demonstration that outcome of interest was not present at start of study* | *** |
| Comparability | |
| *Comparability of cohorts on the basis of the design or analysis* |  |
| Outcome | |
| *Assessment of outcome* | *** |
| *Was follow-up long enough for outcomes to occur* |  |
| *Adequacy of follow up of cohorts* | *** |

Cohort studies with scores of 0-3, 4-6, 7-9 were, respectively, considered as low, moderate, and high quality.

**
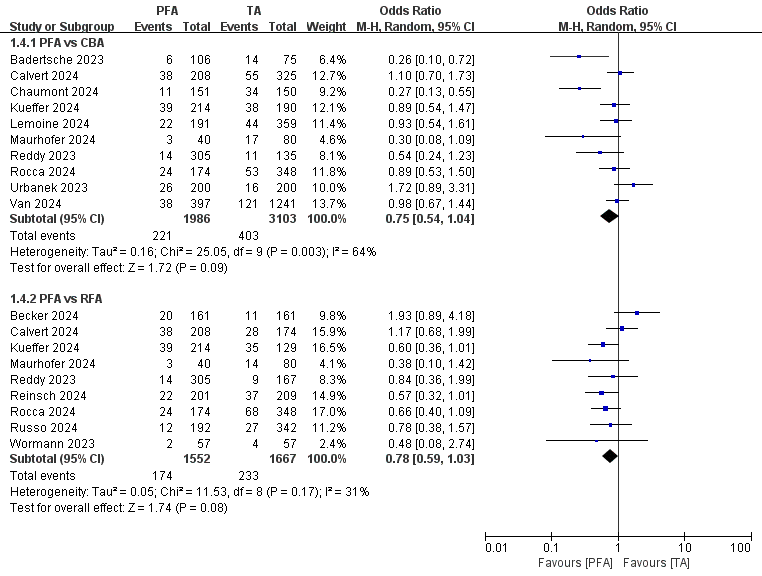
 Figure S1:** The forest plot presents a subgroup analysis comparing the reablation rates after index PFA and TA in the treatment of atrial fibrillation. CI: confidence interval; PFA: pulsed field ablation; TA: thermal ablation

**
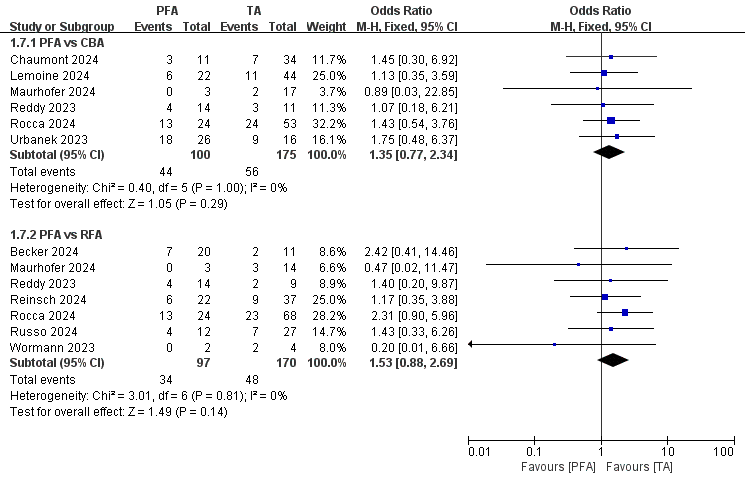
**

**Figure S2:** The forest plot presents a subgroup analysis comparing the lesion durability on a per patient level during the repeat procedure after index PFA and TA. CI: confidence interval; PFA: pulsed field ablation; TA: thermal ablation

**
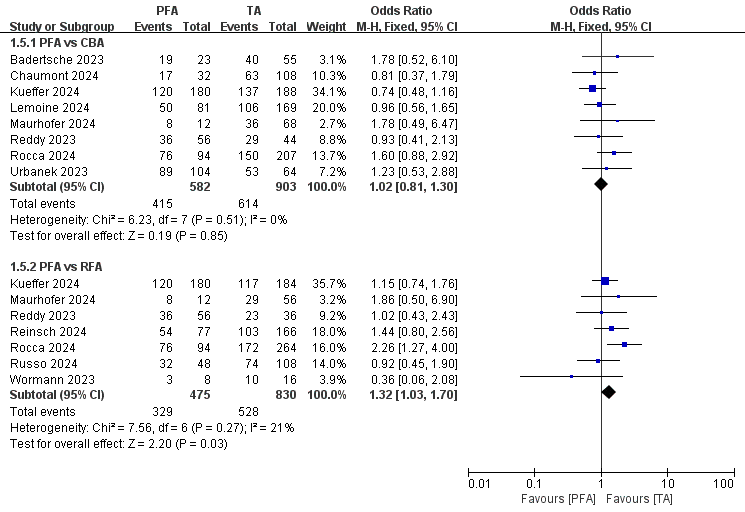
**

**Figure S3:** The forest plot presents a subgroup analysis comparing the lesion durability on a per vein level during the repeat procedure after index PFA and TA. CI: confidence interval; PFA: pulsed field ablation; TA: thermal ablation

(A) (B)


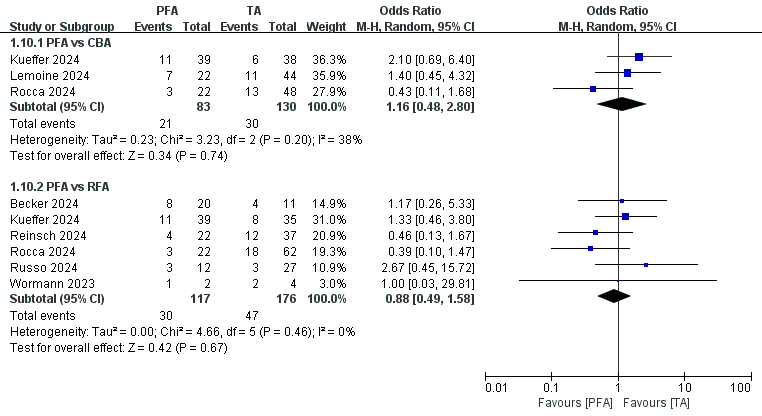

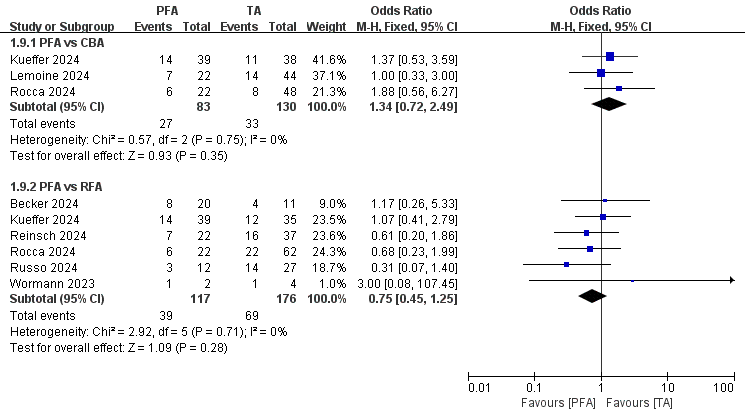


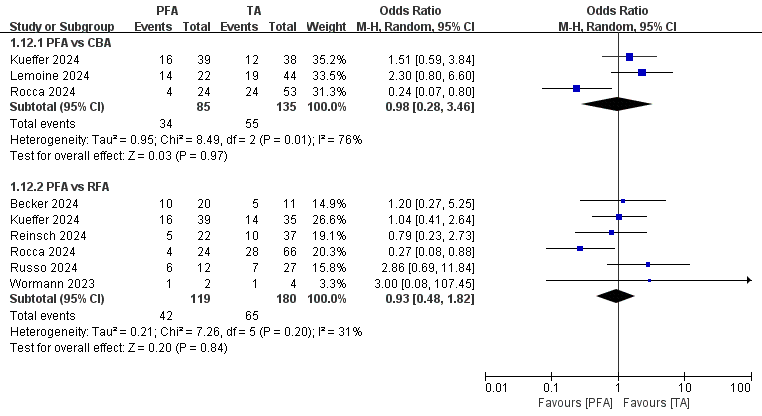
(C) (D)


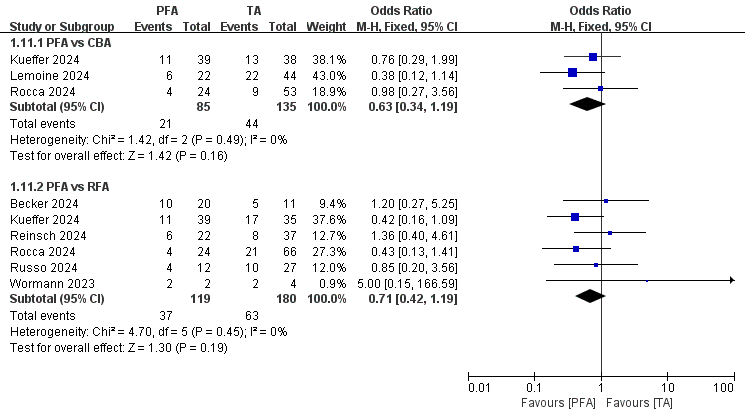


**Figure S4:** The forest plot presents a subgroup analysis comparing the the incidence rates of individual PV reconnection during the repeat procedure after index PFA and TA. (A) left superior PV, (B) left inferior PV, (C) right superior PV, (D) right inferior PV. CI: confidence interval; PFA: pulsed field ablation; PV: pulmonary vein; TA: thermal ablation


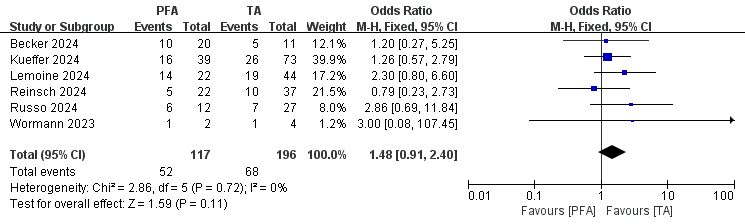


**Figure S5:** The forest plot displays the incidence of right inferior PV during the repeat procedure after index PFA and TA, excluding data from the study conducted by Rocca et al. CI: confidence interval; PFA: pulsed field ablation; PV: pulmonary vein; TA: thermal ablation

1. (B)

**
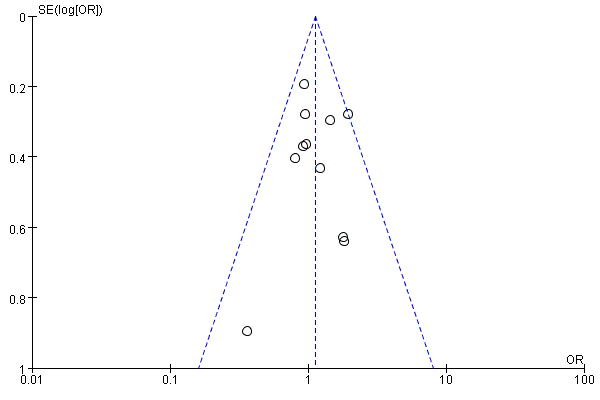

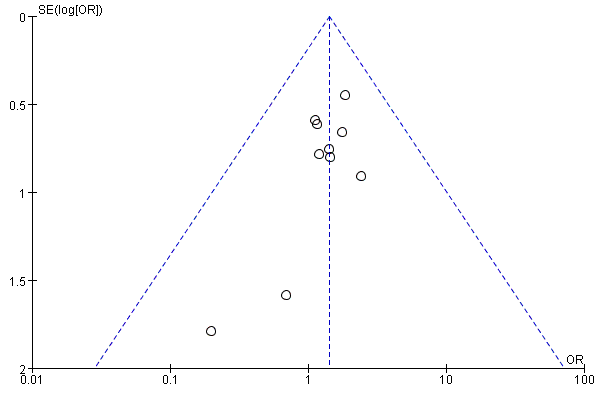
**

**Figure S6** Funnel plots for publication bias. (A) durability PVI per patient, (B) durability PVI per vein. MD: mean difference; OR: odd ratio; PVI: pulmonary vein isolation; SE: standard error
